# Supplementary material for: Spatial patterns of hepatocyte glucose flux revealed by stable isotope tracing and multi-scale microscopy
Source: Nat Commun. 2025 Jul 1;16:5850. doi: 10.1038/s41467-025-60994-w (PMC12219532; doi:10.1038/s41467-025-60994-w)
Supplement: Supplementary file 1 — Supplementary Information [file 41467_2025_60994_MOESM1_ESM.pdf]

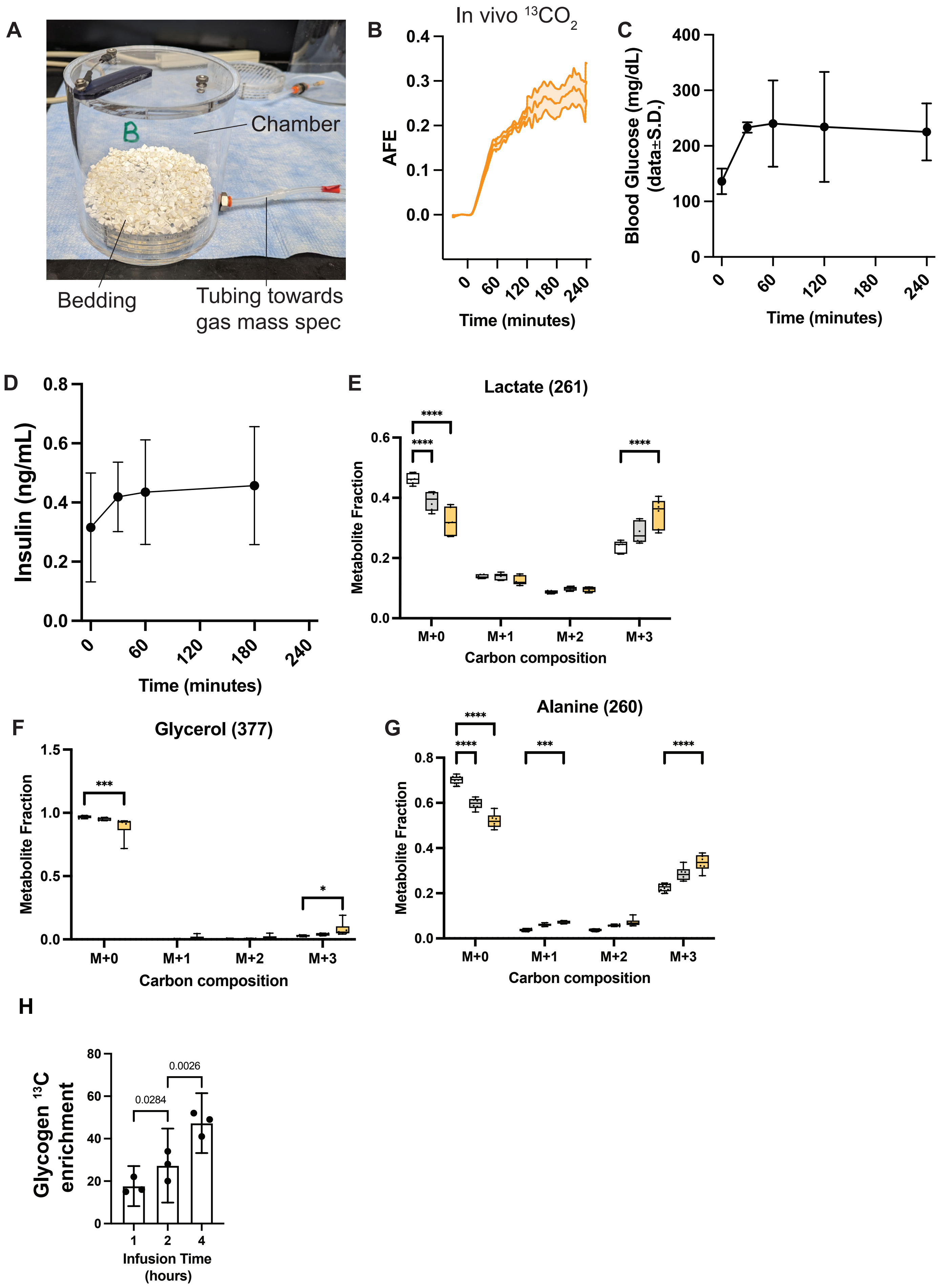

A
Chamber
Bedding
Tubing towards gas mass spec
B
In vivo $^{13}CO_2$
AFE
0.4
0.3
0.2
0.1
0.0
0
60
120
180
240
Time (minutes)
C
Blood Glucose (mg/dL) (data±S.D.)
400
300
200
100
0
0
60
120
180
240
Time (minutes)
D
Insulin (ng/mL)
0.8
0.6
0.4
0.2
0.0
0
60
120
180
240
Time (minutes)
E
Lactate (261)
Metabolite Fraction
0.6
0.4
0.2
0.0
****
****
****
M+0
M+1
M+2
M+3
Carbon composition
F
Glycerol (377)
Metabolite Fraction
1.5
1.0
0.5
0.0
***
*
M+0
M+1
M+2
M+3
Carbon composition
G
Alanine (260)
Metabolite Fraction
0.8
0.6
0.4
0.2
0.0
****
****
***
****
M+0
M+1
M+2
M+3
Carbon composition
H
Glycogen $^{13}C$ enrichment
80
60
40
20
0
0.0284
0.0026
1
2
4
Infusion Time (hours)

**Supplementary Figure 1. In vivo measurements of $^{13}C$-glucose metabolism (A)** Photo of a custom made cylindrical metabolic cage for *in vivo* glucose oxidation measurements. **(B)** Expelled $^{13}CO_2$ (in parts per million (ppm)) in mice continuously infused with 40mg/min/kg for up to 4 hours. **(C-D)** Circulating glucose and insulin levels in mice infused with 40mg/min/kg for up to 4 hours. **(E-G)** GC-MS analysis to determine the fractional $^{13}C$ enrichment of lactate, glycerol, and alanine. Each dot represents an animal. **(H)** $^{13}C$ enrichment accessed using GC-MS of isolated glycogen extracted from [U-$^{13}C$]-glucose-labelled mice after 1, 2, or 4 hours of continuous infusion with 40min/mg/kg of total body mass. P values are shown. In (E-G), **** p<0.001. p values are shown in (H).

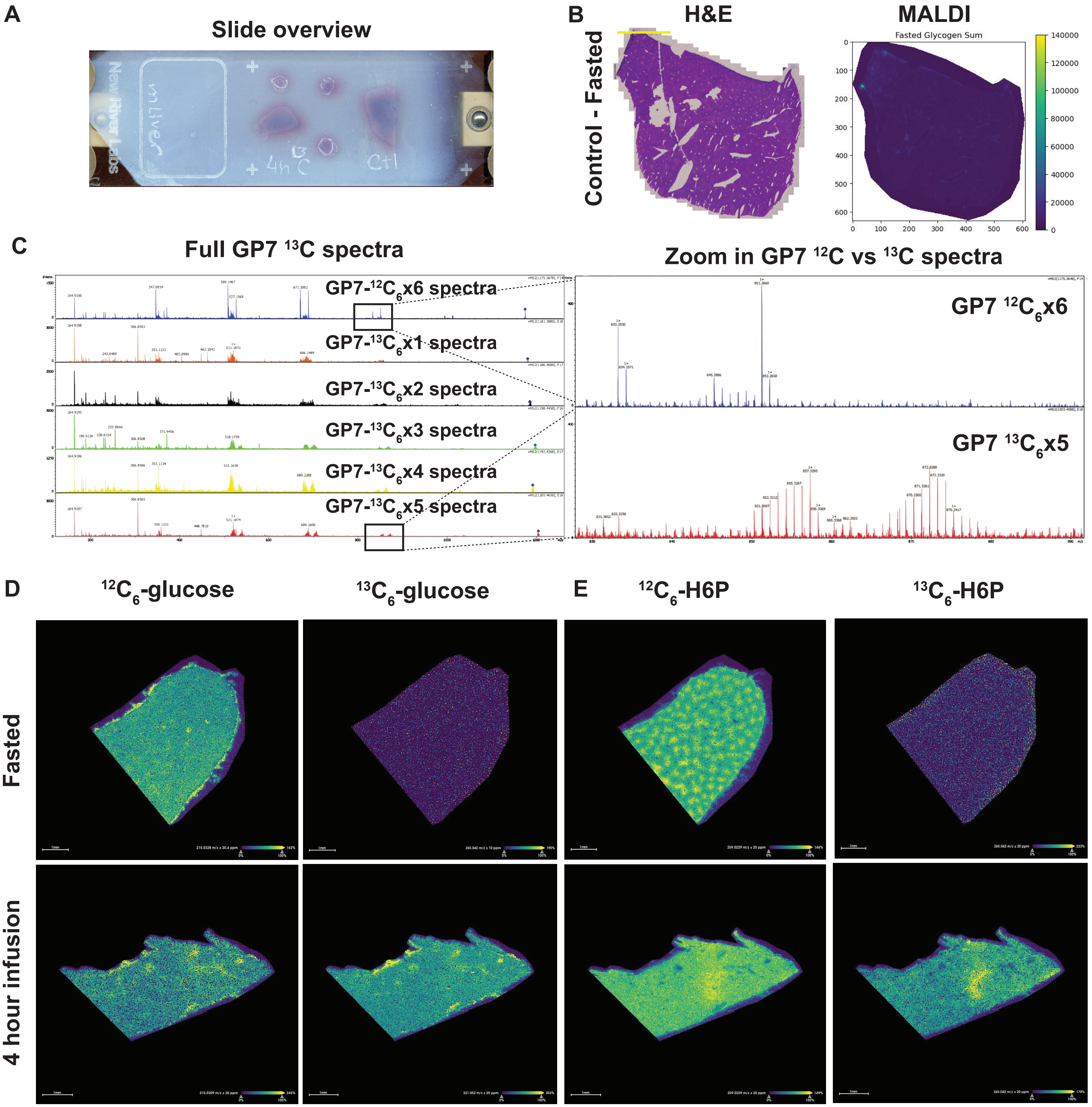
A
Slide overview
B
Control - Fasted
H&E
MALDI
Fasted Glycogen Sum
C
Full GP7 $^{13}C$ spectra
GP7-$^{12}C_6$x6 spectra
GP7-$^{13}C_6$x1 spectra
GP7-$^{13}C_6$x2 spectra
GP7-$^{13}C_6$x3 spectra
GP7-$^{13}C_6$x4 spectra
GP7-$^{13}C_6$x5 spectra
Zoom in GP7 $^{12}C$ vs $^{13}C$ spectra
GP7 $^{12}C_6$x6
GP7 $^{13}C_6$x5
D
$^{12}C_6$-glucose
$^{13}C_6$-glucose
E
$^{12}C_6$-H6P
$^{13}C_6$-H6P
Fasted
4 hour infusion

**Supplementary Figure 2. Imaging mass spectrometry of $^{13}C$-labelled liver sections. (A)** Representative brightfield microscopy image of liver tissue sections mounted on a slide for MALDI-MS imaging. **(B)** Representative images of hematoxylin and eosin (H&E) staining and total glycogen content map measured using MALDI-MS of a mouse liver section fasted overnight and without infusion of [U-$^{13}C_6$]-glucose. **(C)** Snapshot of glycogen mass spectrometry (MS) fragmentation spectra for glucose polymer (GP) with 7 glucose molecules (GP7). Zoom inset shows the spectra of $^{12}C$ and $^{13}C$-labelled GP7. **(D-E)** Liver MALDI-MS images from fasted or [U-$^{13}C$]-glucose-infused (40mg/min/kg) mice and detecting $^{12}C$ or $^{13}C$-labelled glucose and hexose-6-phosphate (H6P) molecules.

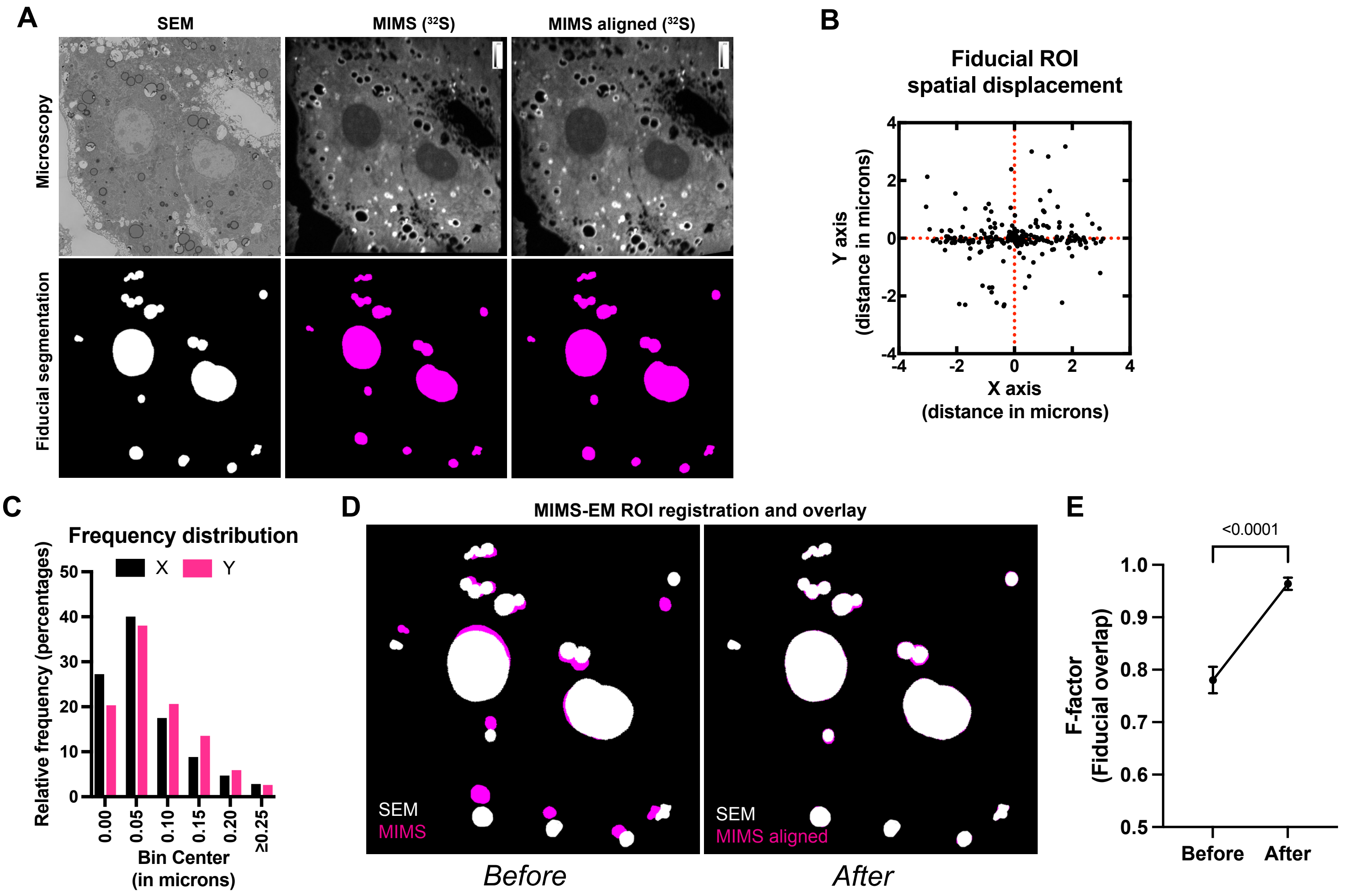
A
SEM
MIMS (32S)
MIMS aligned (32S)
Microscopy
Fiducial segmentation
B
Fiducial ROI
spatial displacement
Y axis
(distance in microns)
X axis
(distance in microns)
C
Frequency distribution
X
Y
Relative frequency (percentages)
Bin Center
(in microns)
D
MIMS-EM ROI registration and overlay
SEM
MIMS
SEM
MIMS aligned
Before
After
E
<0.0001
F-factor
(Fiducial overlap)
Before
After

**Supplementary Figure 3. MIMS-EM imaging of $^{13}C$-labelled hepatocytes. (A)** MIMS-EM registration panels. Raw or aligned MIMS, and SEM data are shown on the top row. MIMS images of the stable isotope $^{32}S$ are shown. Bottom row, segmentation of fiducial markers in SEM and MIMS datasets both before and after MIMS-EM registration. **(B)** Grid graph displaying the displacement of fiducial markers after the image registration process. **(C)** Histogram distribution showing the relative frequency of points in X and Y (plot in the y-axis) and their total spatial displacement in both X and Y axis (bins, in microns, plotted in the x-axis). **(D)** Overlay image of segmented fiducial regions of interest (ROIs) in a representative SEM (white) and MIMS (magenta) dataset before and after registration. **(E)** *F-score* calculated to quantify the overlap of individual SEM and MIMS ROI objects before and after the MIMS-EM image registration process.

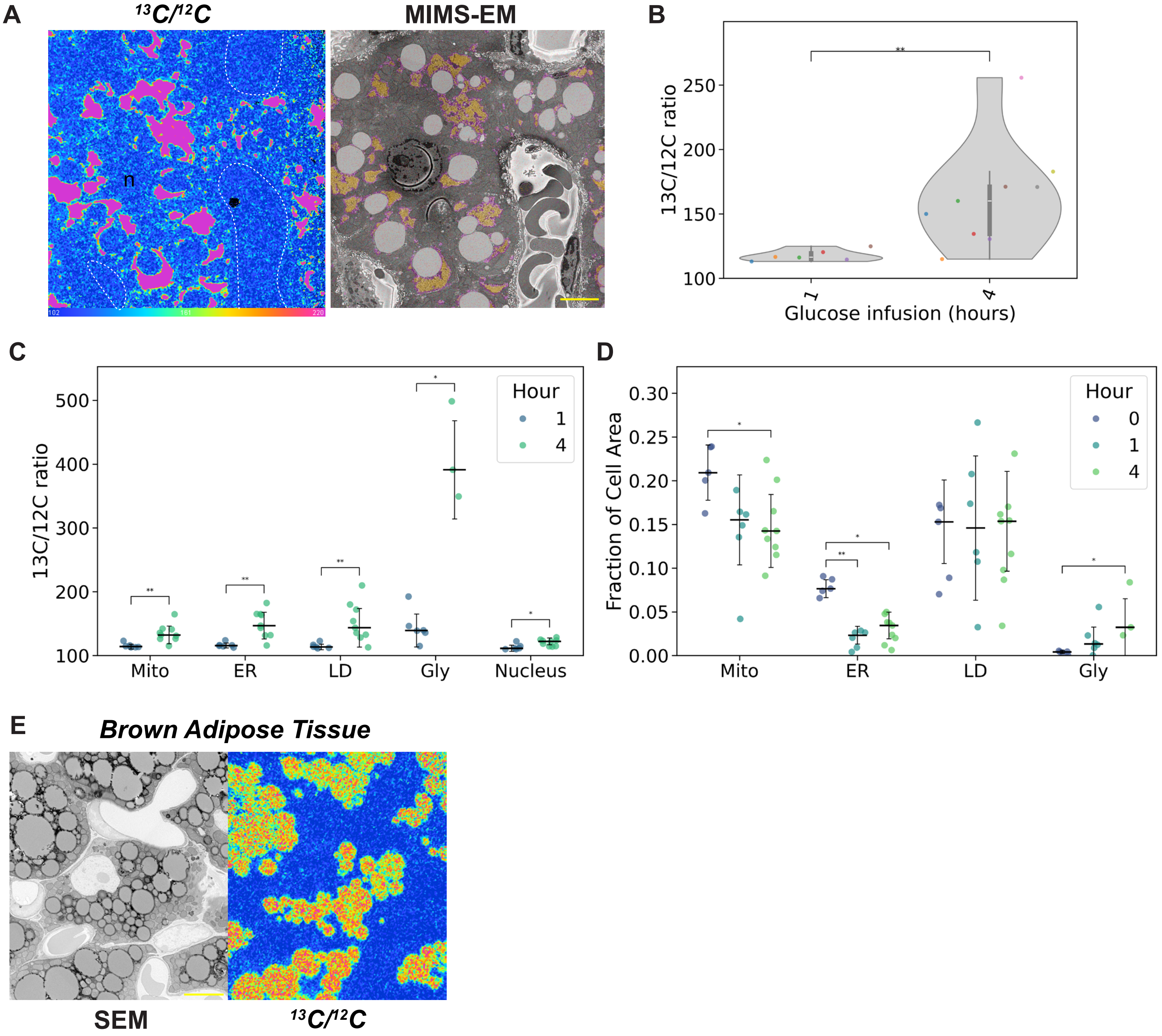
A
$^{13}C/^{12}C$
MIMS-EM
n
102
161
220
B
13C/12C ratio
Glucose infusion (hours)
C
13C/12C ratio
Hour
Mito
ER
LD
Gly
Nucleus
D
Fraction of Cell Area
Hour
Mito
ER
LD
Gly
E
Brown Adipose Tissue
SEM
$^{13}C/^{12}C$

**Supplementary Figure 4. MIMS-EM imaging of mouse hepatocytes.** **(A)** Correlated $^{13}C$-to-$^{12}C$ ($^{13}C/^{12}C$) and SEM images acquired using MIMS-EM of hepatocytes from mice continuously infused with 40mg/min/kg of [U-$^{13}C$]-glucose for 2 hours. Scale bar, 5 microns. **(B)** Mouse averages for hepatocyte $^{13}C/^{12}C$ ratios. **(C)** Mouse averages for hepatocyte organelle type $^{13}C/^{12}C$ ratios. **(D)** Mouse averages for hepatocyte organelle cell fraction. **(E)** Representative MIMS-EM images displaying the $^{13}C/^{12}C$ levels in brown adipocytes from mice infused with 40mg/min/kg of [U-$^{13}C$]-glucose for 4 hours. In (C-D), ** $p<0.01$ and *$p<0.05$ using One-way ANOVA with Dunns test.

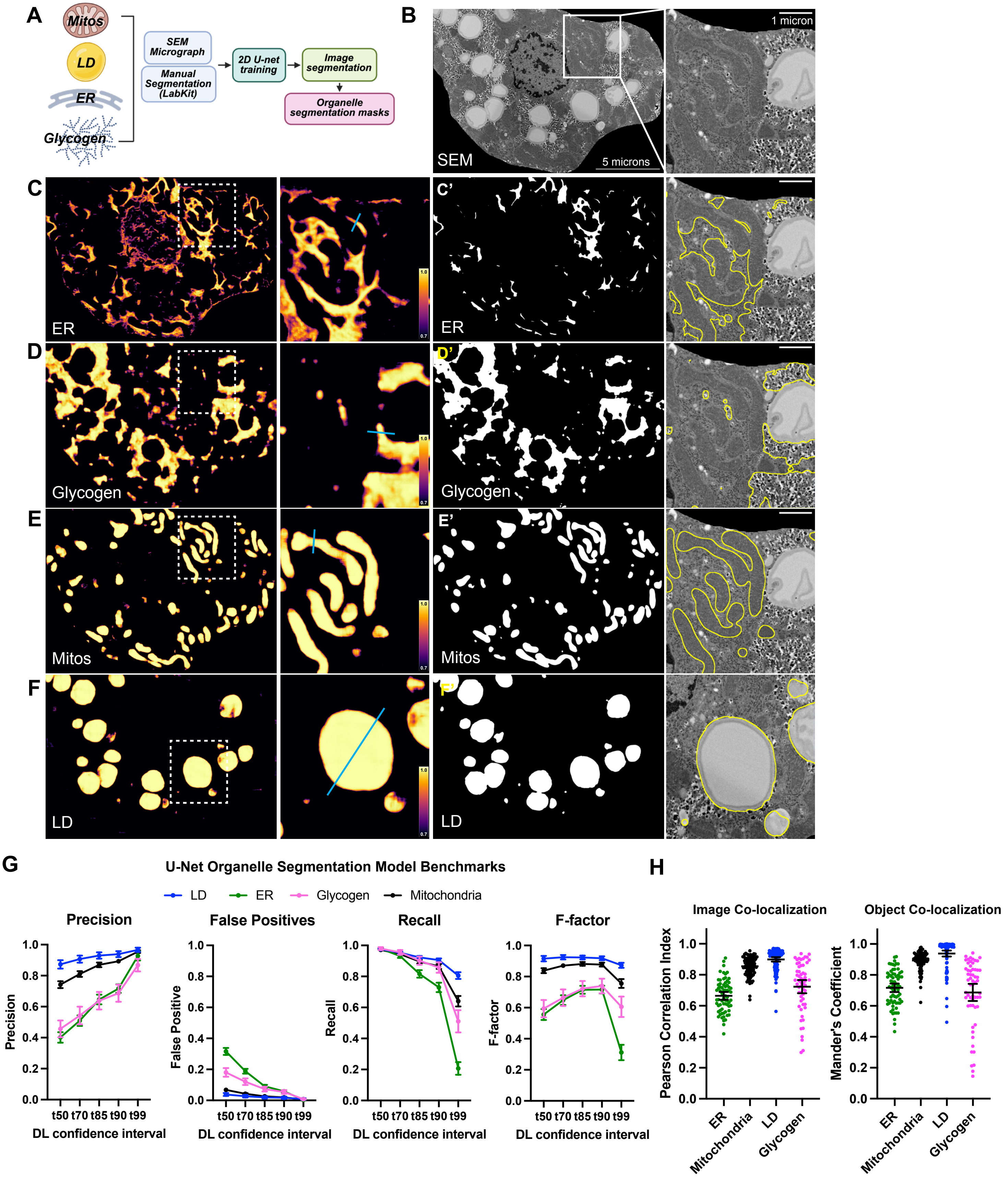
A
Mitos
LD
ER
Glycogen
SEM Micrograph
Manual Segmentation (LabKit)
2D U-net training
Image segmentation
Organelle segmentation masks
B
SEM
5 microns
1 micron
C
ER
C'
ER
D
Glycogen
D'
Glycogen
E
Mitos
E'
Mitos
F
LD
F'
LD
G
U-Net Organelle Segmentation Model Benchmarks
LD
ER
Glycogen
Mitochondria
Precision
False Positives
Recall
F-factor
t50 t70 t85 t90 t99
DL confidence interval
H
Image Co-localization
Object Co-localization
Pearson Correlation Index
Mander's Coefficient
ER
Mitochondria
LD
Glycogen

**Supplementary Figure 5. Machine learning-based segmentation of hepatocyte organelles.** **(A)** Illustration of our organelle segmentation pipeline. A sub-set of SEM images are manually segmented to and used to train a 2D U-net model. This organelle-specific model is applied to SEM micrographs to create organelle segmentation masks that are used for downstream data analysis. Figure created using biorender.com. **(B)** SEM micrograph of a single hepatocyte. Right, zoomed-in region of the cell marked by the white dashed square in (B) to highlight clustered mitochondria, lipid droplet, and glycogen. Cell from a mouse infused with 40mg/min/kg of [U-$^{13}$C]-glucose for 4 hours. **(C-F and C'-F')** Pixel classification confidence maps created by 2D U-nets trained to segment endoplasmic reticulum (ER), glycogen, mitochondria (Mitos), and lipid droplets (LDs), respectively. Here, each pixel receives a score of 0 to 1, where 1 indicates 100% confidence that pixel is correctly classified by a given DL model. In (C-F), raw data from DL-segmentation of the cell shown in (B), including zoomed-in region. Color scale bar represents the segmentation confidence interval from 70-to-100% model confidence. In (C'-F'), binarized organelle segmentation masks after 2D U-net segmentation and image processing and overlay on SEM data. Overlays are shown for the zoomed in quadrant highlighted in (B). **(G)** Performance benchmark indexes for our trained deep-learning image segmentation models. Precision, rate of false positives, object recall, and f-score are shown for different levels of confidence thresholding (t) intervals, from 50-to-95% confidence (t50 to t95). For all our analyzes, we chose a t value of 90%. **(H)** Pearson's and Mander's co-localization indexes quantifying the overlap between manual and 2D U-net segmentation pipelines of the same image or object, respectively.

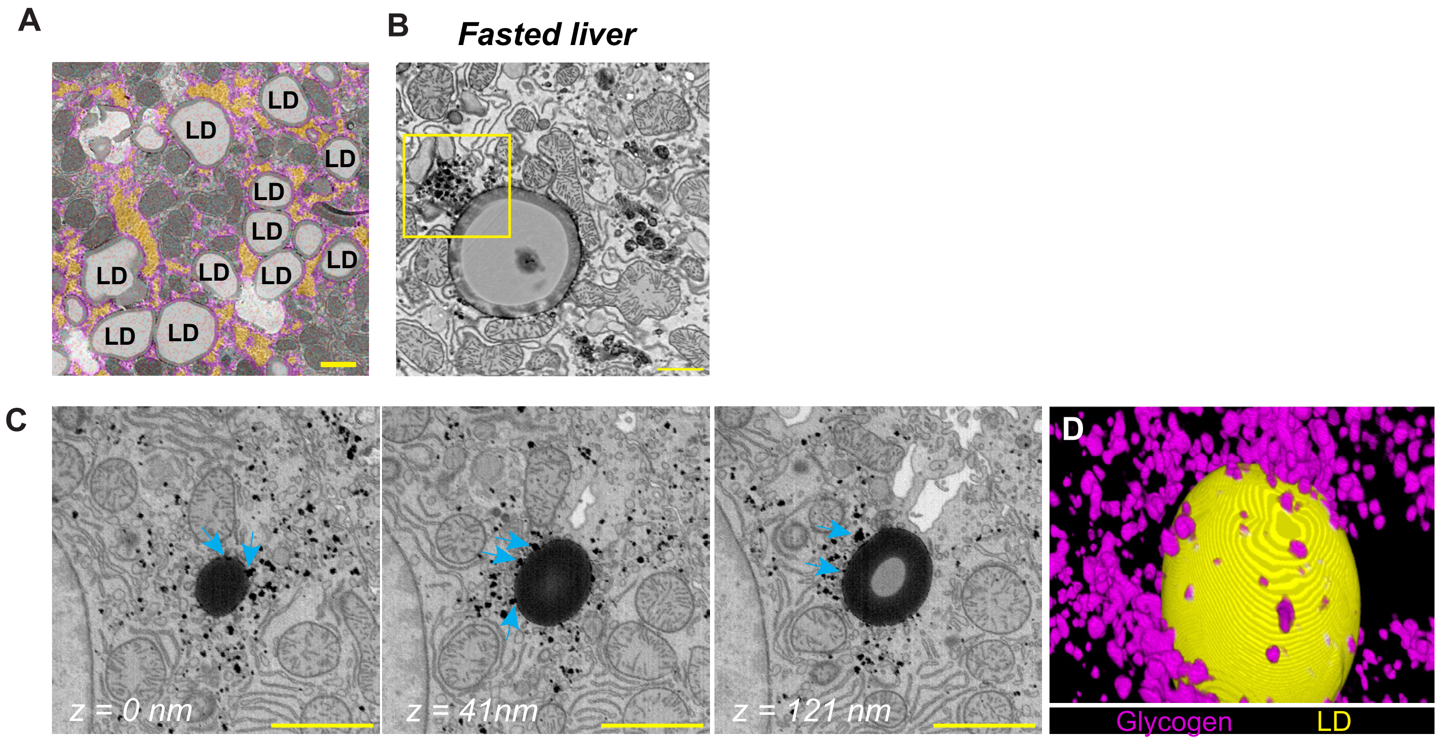
A
LD
LD
LD
LD
LD
LD
LD
LD
LD
LD
LD
B
Fasted liver
C
z = 0 nm
z = 41nm
z = 121 nm
D
Glycogen
LD

**Supplementary Figure 6. MIMS-EM imaging of glucose $^{13}C$ enrichment around lipid droplets (LDs). (A)** Representative zoomed in MIMS-EM image of the hepatocyte shown in Supplemental Figure 3B. Lipid droplets (LD) are annotated. Scale bar, 1 micron. **(B)** Representative scanning electron microscopy (SEM) image of a mouse hepatocyte after overnight fasting. Yellow bounding box highlights small glycogen clusters next to a lipid droplet (LD). **(C)** Serial slices extracted from a previously published study with a volumetric EM dataset centered in a lipid droplet. Relative distance in the z-axis from the first section are shown in white. Blue arrows indicate glycogen depots in the periphery of the lipid droplet. **(D)** 3D reconstruction rendering of the volume shown in (C).

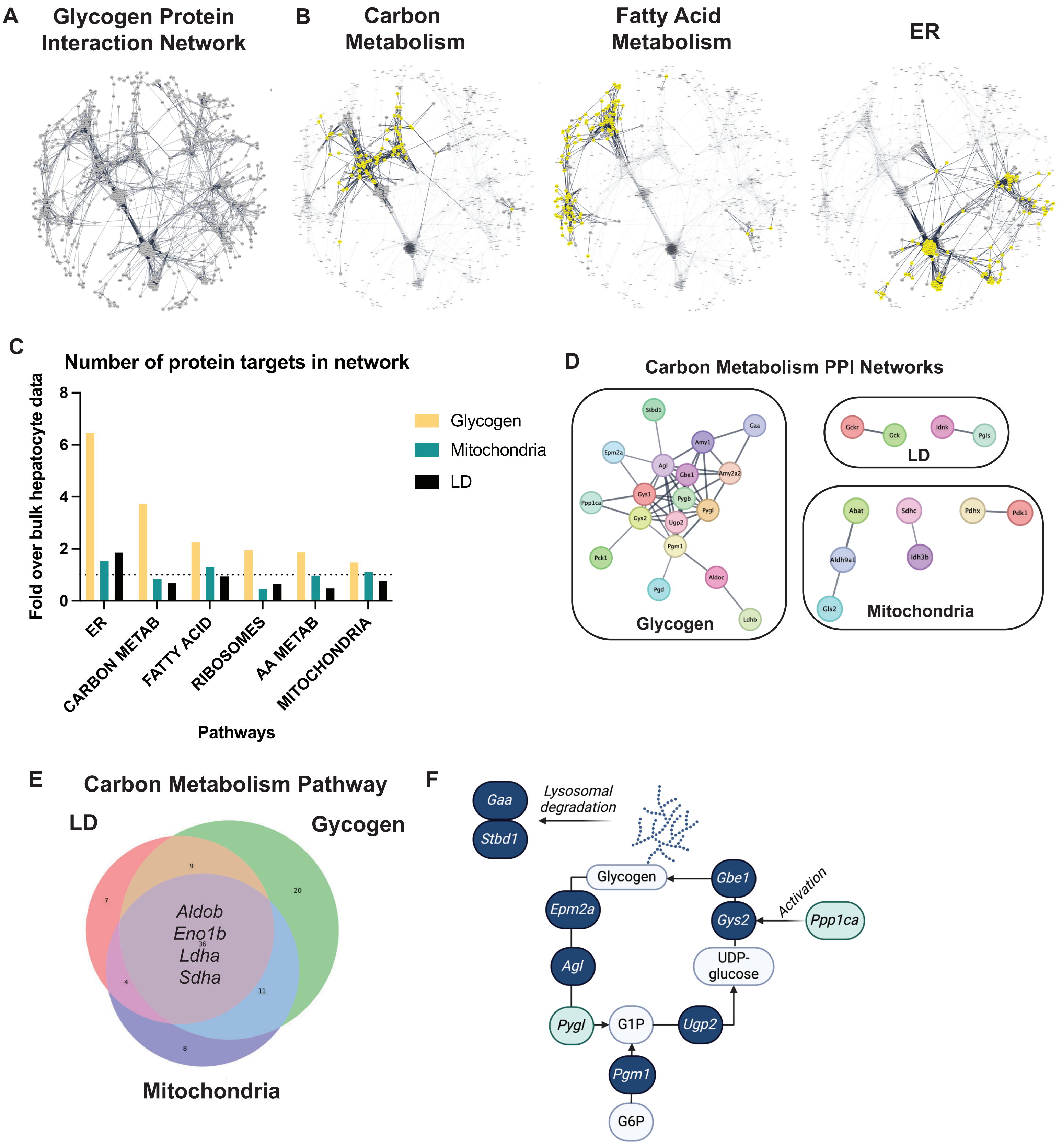
A
Glycogen Protein Interaction Network
B
Carbon Metabolism
Fatty Acid Metabolism
ER
C
Number of protein targets in network
Fold over bulk hepatocyte data
8
6
4
2
0
Glycogen
Mitochondria
LD
ER
CARBON METAB
FATTY ACID
RIBOSOMES
AA METAB
MITOCHONDRIA
Pathways
D
Carbon Metabolism PPI Networks
Stbd1
Gaa
Amy1
Epm2a
Agl
Gbe1
Amy2a2
Gys1
Ppp1ca
Pygb
Pygl
Gys2
Ugp2
Pgm1
Pck1
Aldoc
Pgd
Ldhb
Glycogen
Gckr
Gck
Idnk
Pgls
LD
Abat
Sdhc
Pdhx
Pdk1
Aldh9a1
Idh3b
Gls2
Mitochondria
E
Carbon Metabolism Pathway
LD
Gycogen
9
7
20
Aldob
Eno1b
36
Ldha
Sdha
4
11
8
Mitochondria
F
Gaa
Stbd1
Lysosomal degradation
Glycogen
Gbe1
Epm2a
Gys2
Activation
Ppp1ca
UDP-glucose
Agl
Pygl
G1P
Ugp2
Pgm1
G6P

**Supplementary Figure 7. STRING DB analysis of hepatocyte proteins. (A)** Reconstructed protein-protein interaction network of glycogen-enriched proteins detected using mass spectrometry of isolated glycogen analyzed from [47]. Networks were built using the top 2000 most abundant proteins and queried using StringDB and visualized using Cytoscape. Each node represents a protein in the network. **(B)** Yellow nodes represent proteins significantly enriched in carbon metabolism, fatty acid metabolism, or ER-associated pathways. **(C)** Relative enrichment of proteins found in the isolated glycogen, mitochondria, or lipid droplet (LD) mass spectrometry datasets. Proteins were clustered using StringDB clustering to identify proteins linked to ER, carbon metabolism, fatty acid metabolism, ribosomes, amino acid (AA) metabolism, and mitochondria-related pathways. Data reflects a fold change in enrichment for each pathway using data acquired from bulk hepatocyte proteomics as a benchmark. Dotted line marks a fold enrichment of 1. **(D)** Reconstructed protein-protein interaction network of proteins enriched in LD, glycogen, and mitochondria datasets. Protein names are shown inside nodes. **(E)** Venn diagram illustrating the overlap of proteins identified in glycogen, mitochondria, or LD-isolated fractions. Common proteins are highlighted in the middle. Numbers represent number of proteins inside each unique or overlapping dataset. **(F)** Schematic illustration of the metabolites (shown in grey) and proteins involved in the glycogenesis pathway. All proteins were found in the glycogen dataset. Proteins in aquamarine color are known to be in the endoplasmic reticulum (ER).

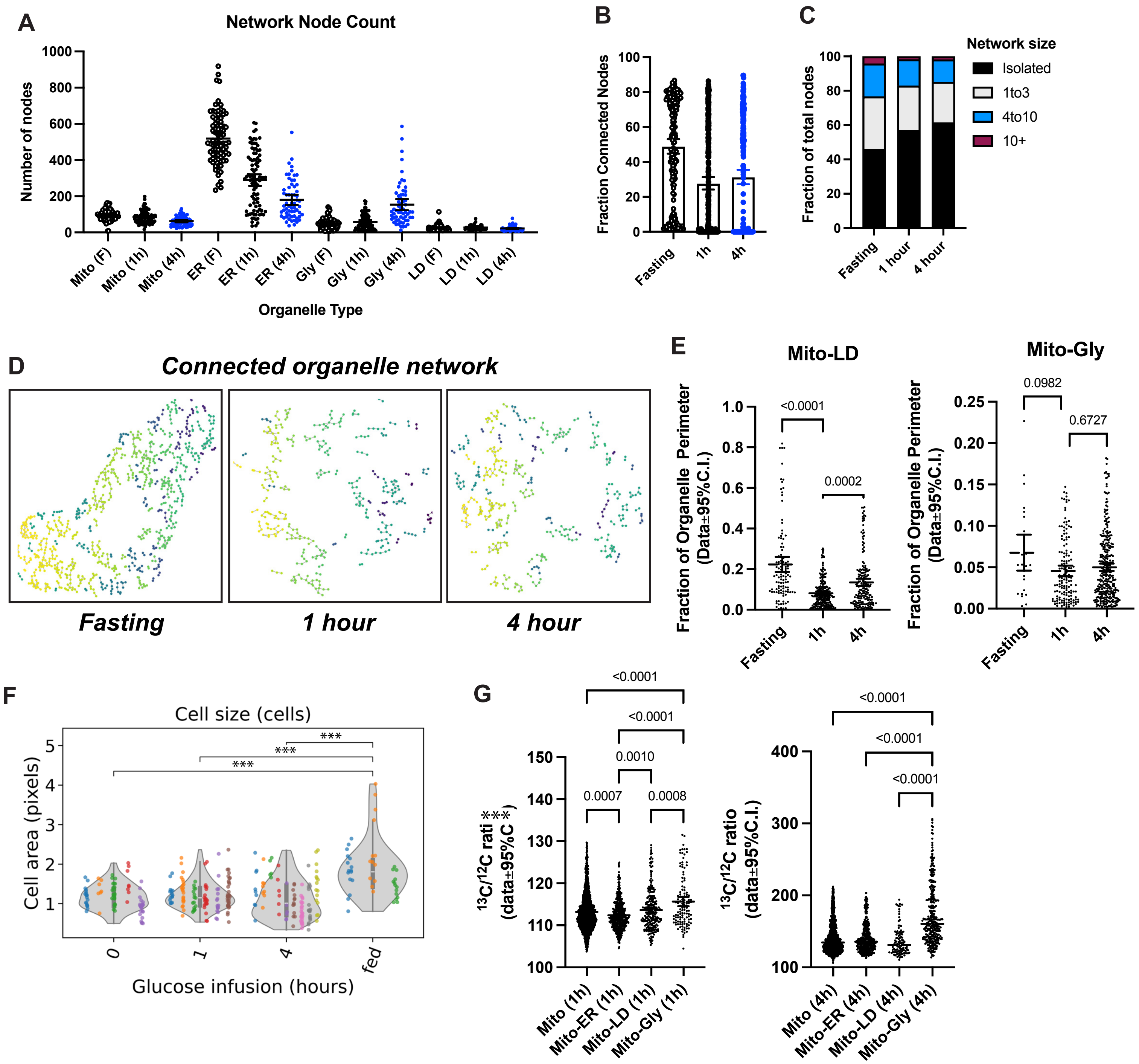

A
Network Node Count
Number of nodes
Organelle Type
Mito (F)
Mito (1h)
Mito (4h)
ER (F)
ER (1h)
ER (4h)
Gly (F)
Gly (1h)
Gly (4h)
LD (F)
LD (1h)
LD (4h)
B
Fraction Connected Nodes
Fasting
1h
4h
C
Fraction of total nodes
Network size
Isolated
1to3
4to10
10+
Fasting
1 hour
4 hour
D
Connected organelle network
Fasting
1 hour
4 hour
E
Mito-LD
Mito-Gly
Fraction of Organelle Perimeter (Data±95%C.I.)
<0.0001
0.0002
0.0982
0.6727
F
Cell size (cells)
Cell area (pixels)
Glucose infusion (hours)
***
0
1
4
fed
G
13C/12C rati *** (data±95%C.I.)
13C/12C ratio (data±95%C.I.)
<0.0001
0.0010
0.0007
0.0008
Mito (1h)
Mito-ER (1h)
Mito-LD (1h)
Mito-Gly (1h)
Mito (4h)
Mito-ER (4h)
Mito-LD (4h)
Mito-Gly (4h)

**Supplementary Figure 8. Network analysis of hepatocyte organelle connections. (A)** Total number of nodes per cell network and annotated to endoplasmic reticulum (ER), glycogen (Gly), mitochondria (Mito), or lipid droplets (LD) organelles. **(B)** Relative fraction of nodes that have a connection within a 500 nanometer (nm) distance radius. **(C)** Relative fraction of network nodes that either lack connections (i.e., isolated), or have between 1-to-3 (1to3), 4-to-10 (4to10), or more than 10 (10+) node connections. **(D)** Representative organelle network maps displaying connected nodes only. **(E)** Percentage of mitochondrial perimeter covered by LD, or glycogen contact sites. **(F)** Hepatocyte cell size in mice after overnight fasting, 1 or 4 hours of glucose infusion, or during a fed state. **(G)** $^{13}C/^{12}C$ ratio in isolated mitochondria (Mito) or mitochondria with ER, LD, or glycogen (Gly) contacts. In (E), p values are shown. In (F), *** $p<0.001$, and different colors represent data from individual animals. One-way ANOVA with Dunns test post-hoc was used in (F) or Dunns or Kruskal-Wallis test was used in (E and G).

**Supplementary Table 1. TM Sprayer parameters for application of enzyme**

| TM Sprayer M3 | |
|---|---|
| Enzyme | Isoamylase |
| Solvent | $H_2O$ |
| Temp (°C) | 45 |
| # Passes (Count) | 15 |
| Flow Rate (ml/min) | 0.15 |
| Velocity (mm/min) | 900 |
| Track Spacing (mm) | 3 |
| Pattern | CC |
| Pressure (psi) | 10 |
| Gas Flow Rate (l/min) | 2 |
| Drying Time (s) | 0 |
| Nozzle Hight (mm) | 40 |

**Supplementary Table 2. Additional MALDI IMS instrument parameters**

| TM Sprayer M5 | |
|---|---|
| Matrix | CHCA |
| Solvent | 1:1 ACN: $H_2O$ (0.1% TFA) |
| Temp (°C) | 72.5 |
| # Passes (Count) | 24 |
| Concentration (mg/ml) | 5 |
| Flow Rate (ml/min) | 0.05 |
| Velocity (mm/min) | 1300 |
| Track Spacing (mm) | 3 |
| Pattern | CC |
| Pressure (psi) | 10 |
| Gas Flow Rate (l/min) | 2 |
| Drying Time (s) | 2 |
| Nozzle Hight (mm) | 40 |

**Supplementary Table 3. Additional MALDI IMS instrument parameters**

| Transfer | |
|---|---|
| Deflection 1 Delta | (-)80.0 V |
| Funnel 1 RF | 350.0 Vpp |
| isCID Energy | 10.0 eV Vpp |
| Funnel 2 RF | 500.0 Vpp |
| Multipole RF | 500.0 Vpp |
| Collision Cell | |
| Collision Energy | 25.0 eV |
| Collision RF | 3000.0 Vpp |
| Transfer Time | 110 µs |
| Pre Pulse Storage | 15.0 µs |

Quadrupole

| | |
|---|---|
| Ion Energy | 25.0 eV |
| Low Mass | m/z 500.00 |
